# Supplementary material for: Resolving the full spectrum of human genome variation using Linked-Reads
Source: Genome Res. 2019 Apr;29(4):635–45. doi: 10.1101/gr.234443.118 (PMC6442396; doi:10.1101/gr.234443.118)
Supplement: Supplemental Material [file supp_gr.234443.118_Supplemental_longranger-2.2.zip › longranger-2.2.tar.bz2!/lr2.2-public/lib/stan/cmdstan/stan/doc/api/html/functions_p.html]

Stan: Class Members


|  |  |
| --- | --- |
|  | Stan  2.5.0  probability, sampling & optimization |


- Main Page
- Related Pages
- Namespaces
- Classes
- Files

- Class List
- Class Index
- Class Hierarchy
- Class Members

- All
- Functions
- Variables
- Typedefs
- Enumerator
- Related Functions

- \_
- a
- b
- c
- d
- e
- f
- g
- h
- i
- j
- k
- l
- m
- n
- o
- p
- q
- r
- s
- t
- u
- v
- w
- x
- y
- z
- ~


All Classes Namespaces Files Functions Variables Typedefs Enumerations Enumerator Friends Macros Pages

Here is a list of all class members with links to the classes they belong to:

### - p -

- p
  : stan::mcmc::ps\_point
- param\_name()
  : stan::mcmc::chains< RNG >
- param\_names()
  : stan::mcmc::chains< RNG >
- param\_range\_i()
  : stan::model::prob\_grad
- param\_ranges\_i\_\_
  : stan::model::prob\_grad
- param\_var\_decls\_r
  : stan::gm::program\_grammar< Iterator >
- parameter\_decl\_
  : stan::gm::program
- params\_r()
  : stan::optimization::BFGSLineSearch< M, QNUpdateType, Scalar, DimAtCompile >
- parse()
  : stan::io::stan\_csv\_reader
- parse\_args()
  : stan::gm::argument
  , stan::gm::argument\_parser
  , stan::gm::categorical\_argument
  , stan::gm::list\_argument
  , stan::gm::singleton\_argument< T >
  , stan::gm::unvalued\_argument
- partials\_
  : stan::agrad::stored\_gradient\_vari
- partials\_vari()
  : stan::agrad::partials\_vari
- phi()
  : stan::mcmc::base\_hamiltonian< M, P, BaseRNG >
  , stan::mcmc::dense\_e\_metric< M, BaseRNG >
  , stan::mcmc::diag\_e\_metric< M, BaseRNG >
  , stan::mcmc::unit\_e\_metric< M, BaseRNG >
- positive\_ordered()
  : stan::io::reader< T >
- positive\_ordered\_constrain()
  : stan::io::reader< T >
- positive\_ordered\_decl\_r
  : stan::gm::var\_decls\_grammar< Iterator >
- positive\_ordered\_unconstrain()
  : stan::io::writer< T >
- positive\_ordered\_var\_decl()
  : stan::gm::positive\_ordered\_var\_decl
- precomp\_v\_vari()
  : stan::agrad::precomp\_v\_vari
- precomputed\_gradients\_vari()
  : stan::agrad::precomputed\_gradients\_vari
- prev\_f()
  : stan::optimization::BFGSMinimizer< FunctorType, QNUpdateType, Scalar, DimAtCompile >
- prev\_g()
  : stan::optimization::BFGSMinimizer< FunctorType, QNUpdateType, Scalar, DimAtCompile >
- prev\_p()
  : stan::optimization::BFGSMinimizer< FunctorType, QNUpdateType, Scalar, DimAtCompile >
- prev\_step\_size()
  : stan::optimization::BFGSMinimizer< FunctorType, QNUpdateType, Scalar, DimAtCompile >
- prev\_x()
  : stan::optimization::BFGSMinimizer< FunctorType, QNUpdateType, Scalar, DimAtCompile >
- print()
  : stan::gm::argument
  , stan::gm::argument\_parser
  , stan::gm::categorical\_argument
  , stan::gm::list\_argument
  , stan::gm::unvalued\_argument
  , stan::gm::valued\_argument
  , stan::io::cmd\_line
- print\_help()
  : stan::gm::argument
  , stan::gm::argument\_parser
  , stan::gm::categorical\_argument
  , stan::gm::list\_argument
  , stan::gm::unvalued\_argument
  , stan::gm::valued\_argument
- print\_statement()
  : stan::gm::print\_statement
- print\_statement\_r
  : stan::gm::statement\_grammar< Iterator >
- print\_usage()
  : stan::gm::argument\_parser
- print\_valid()
  : stan::gm::list\_argument
  , stan::gm::singleton\_argument< T >
  , stan::gm::valued\_argument
- print\_value()
  : stan::gm::list\_argument
  , stan::gm::singleton\_argument< T >
  , stan::gm::valued\_argument
- printable()
  : stan::gm::printable
- printable\_
  : stan::gm::printable
- printable\_r
  : stan::gm::statement\_grammar< Iterator >
- printable\_string\_r
  : stan::gm::statement\_grammar< Iterator >
- printable\_t
  : stan::gm::printable
- printable\_visgen()
  : stan::gm::printable\_visgen
- printables\_
  : stan::gm::print\_statement
  , stan::gm::reject\_statement
- prob()
  : stan::io::reader< T >
- prob\_constrain()
  : stan::io::reader< T >
- prob\_grad()
  : stan::model::prob\_grad
- prob\_unconstrain()
  : stan::io::writer< T >
- probe\_args()
  : stan::gm::argument
  , stan::gm::argument\_probe
  , stan::gm::categorical\_argument
  , stan::gm::list\_argument
  , stan::gm::singleton\_argument< T >
- program()
  : stan::gm::program
- program\_grammar()
  : stan::gm::program\_grammar< Iterator >
- program\_r
  : stan::gm::program\_grammar< Iterator >
- promote()
  : stan::math::promoter< F, T >
  , stan::math::promoter< Eigen::Matrix< F, R, C >, Eigen::Matrix< T, R, C > >
  , stan::math::promoter< Eigen::Matrix< T, R, C >, Eigen::Matrix< T, R, C > >
  , stan::math::promoter< std::vector< F >, std::vector< T > >
  , stan::math::promoter< std::vector< T >, std::vector< T > >
  , stan::math::promoter< T, T >
- promote\_to()
  : stan::math::promoter< F, T >
  , stan::math::promoter< Eigen::Matrix< F, R, C >, Eigen::Matrix< T, R, C > >
  , stan::math::promoter< Eigen::Matrix< T, R, C >, Eigen::Matrix< T, R, C > >
  , stan::math::promoter< std::vector< F >, std::vector< T > >
  , stan::math::promoter< std::vector< T >, std::vector< T > >
  , stan::math::promoter< T, T >
- ps\_point()
  : stan::mcmc::ps\_point
- ps\_point\_test
  : stan::mcmc::ps\_point


---

[ Stan Home Page ]

*© 2011–2014,
Stan Development Team.*
